# Transcriptional Activation of the Cholecystokinin Gene by DJ-1 through Interaction of DJ-1 with RREB1 and the Effect of DJ-1 on the Cholecystokinin Level in Mice

**DOI:** 10.1371/journal.pone.0078374

**Published:** 2013-11-05

**Authors:** Takuya Yamane, Sayaka Suzui, Hirotake Kitaura, Kazuko Takahashi-Niki, Sanae M. M. Iguchi-Ariga, Hiroyoshi Ariga

**Affiliations:** 1 Graduate School of Pharmaceutical Sciences, Hokkaido University, Sapporo, Japan; 2 Graduate School of Agriculture, Hokkaido University, Sapporo, Japan; University of Hong Kong, Hong Kong

## Abstract

*DJ-1* is an oncogene and also causative gene for familial Parkinson’s disease. DJ-1 has multiple functions, including transcriptional regulation. DJ-1 acts as a coactivator that binds to various transcription factors, resulting in stimulation or repression of the expression of their target genes. In this study, we found that the cholecystokinin (CCK) gene is a transcriptional target gene for DJ-1. CCK is a peptide hormone and plays roles in contraction of the gallbladder and in promotion of secretion of pancreatic fluid. CCK is co-localized with dopamine in the substantia nigra to regulate release of dopamine. Reduced expression of CCK mRNA was observed in DJ-1-knockdown cells. The Ras-responsive element (RRE) and Sp1 site were essential for promoter activity, and DJ-1 stimulated promoter activity by binding to RRE-binding protein 1 (RREBP1). The complex of DJ-1 with RREB1 but not with Sp1 bound to the RRE. Furthermore, the reduced CCK level in the serum from DJ-1-knockout mice compared to that from wild-type mice was observed. This is the first report showing that DJ-1 participates in peptide hormone synthesis.

## Introduction

The *DJ-1* gene has been identified by us as a novel oncogene that transforms NIH3T3 cells in cooperation with the activated *ras* gene [1] and was later found to be a causative gene for familial Parkinsons disease *park7* [2]. Since a high level of DJ-1 expression was found in various cancers and since the *DJ-1* gene is localized at chromosome 1p36.2–36.3, which is the hot spot for localization of cancer-related genes, it is assumed that expression and function of DJ-1 are deeply correlated to onset of cancers [3]–[5]. DJ-1 has multiple functions, including transcriptional regulation [6]–[15]. DJ-1 binds to various transcription factors, including inhibitors for androgen receptor [6], [7], p53 [8], [13], [15], polypyrimidine tract-binding protein-associated splicing factor (PSF) [9], Keap1, an inhibitor for nuclear factor erythroid-2 related factor 2 (Nrf2) [10], and sterol regulatory element binding protein (SREBP) [16] to modulate their transcriptional activity, resulting in various effects on cell functions.

We previously searched for genes whose expression was changed in DJ-1-knockdown cells compared to that in parental cells by using a DNA microarray, and we identified many candidate genes, including the cholecystokinin (CCK) gene [17]. CCK is a peptide hormone and plays roles in contraction of the gallbladder and in promotion of secretion of pancreatic fluid [18]. CCK also functions in appetite regulation and hypermnesia in the brain [19]. CCK is co-localized with dopamine in the substantia nigra to regulate release of dopamine [20]–[23]. It is therefore of interest to analyze the role of DJ-1 in regulation of CCK expression. In this study, we found by using a cell culture system that DJ-1 stimulates expression of the *CCK* gene at the transcriptional level by association with RREB1 and that the reduced CCK level in the serum from DJ-1-knockout mice compared to that from wild-type mice was observed.

## Materials and Methods

### Cells

NIH3T3 and SY-SY5Y cells were purchased from American Tissue culture collection (ATCC). Establishment of DJ-1-knockdown NIH3T3 (D2) and SH-SY5Y (KD4) cells was described previously [24], [25]. D2 and KD4 cells and their parental NIH3T3 and SH-SY5Y cells were cultured in Dulbecco’s modified Eagle’s medium with 10% calf serum. Knockdown of RREB1 was carried out after transfection of RREB1 siRNA into NIH3T3 cells in 6-well plates by Lipofectamine 2000 (Invitrogen, Carlsbad, CA), and the CCK mRNA level was determined by real-time PCR at 48 hrs after transfection. Nucleotide sequence sequences of upper and lower strands of siRNA were 5′-CAUGAAACCUCCAGGACCATT-3′ and 5′-UGGUCCUGGAGGUUUCAUGTT-3′, respectively.

### RT-PCR and Real-time PCR

Nucleotide sequences of primers and PCR conditions used for RT-PCR and real-time PCR are shown in Table 1. Total RNAs were prepared from cells and subjected to semi-quantitative RT-PCR analyses. After reactions, PCR products were extracted, separated on 1.4% agarose gels, and stained with ethidium bromide. Reverse images of black and white staining are shown. Quantitative RT-PCR (real-time PCR) analyses were carried out as described previously [26].

**Table 1:** Nucleotide sequences of primers and PCR conditions used for RT-PCR and real-time PCR.

Gene	Nucleotide sequence		PCR condition
mCCK	sense-R	5′-GGTAGTCCCTGCAGAAGCTACGG-3′	94°C 4 min, 94°C 30 sec, 62°C 30 sec, 72°C 1 min×30 cycles
	anti-sense-R	5′-TTGGTGTGTATGCTGGGGAGC-3′	
mDJ-1	sense-R	5′-GCTTCCAAAAGAGCTCTGGTCA-3	94°C 4 min, 94°C 30 sec, 58°C 30 sec, 72°C 1 min×30 cycles
	anti-sense-R	5′-GCTCTAGTCTTTGAGAACAAGC-3′	
mACTB	sense-R	5′-CCTAGGCACCAGGGTGTGAT-3′	94°C 4 min, 94°C 30 sec, 58°C 30 sec, 72°C 1 min×30 cycles
	anti-sense-R	5′-GCTCGAAGTCTAGAGCAACA-3′	
hCCK	sense-R	5′-GCGTCCTAATCCAAAAGCCATG-3′	94°C 4 min, 94°C 30 sec, 61°C 30 sec, 72°C 1 min×28 cycles
	anti-sense-R	5′-TGGGTTGGGAGGTTGCTTC-3′	
hDJ-1	sense-R	5′-GGTGCAGGCTTGTAAACATATAAC-3′	94°C 4 min, 94°C 30 sec, 56°C 30 sec, 72°C 1 min×22 cycles
	anti-sense-R	5′-CTCTAAGTGATCGTCGCAGTTCGC-3′	
hACTB	sense-R	5′-CCGACAGGATGCAGAAGCAG-3′	94°C 4 min, 94°C 30 sec, 56°C 30 sec, 72°C 1 min×20 cycles
	anti-sense-R	5′-GTGGGGTGGCTTTTAGGATG-3′	
mCCK	sense-Q	5′-TGGACCCCAGCCATAGAATAA-3′	95°C 10 sec, 95°C 5 sec, 60°C 20 sec×44 cycles
	anti-sense-Q	5′-CCTCATTCCACCTCCTCCAA-3′	
mDJ-1	sense-Q	5′-GCACCGCTTGTTCTCAAAG-3′	95°C 10 sec, 95°C 5 sec, 60°C 20 sec×44 cycles
	anti-sense-Q	5′-TGGCAGGAGCTTGGTAAACT-3′	
mACTB	sense-Q	5′-CCCTAAGGCCAACCGTGAAA-3′	95°C 10 sec, 95°C 5 sec, 60°C 20 sec×44 cycles
	anti-sense-Q	5′-ACGACCAGAGGCATACAGGGA-3′	
hCCK	sense-Q	5′-GCGTCCTAATCCAAAAGCCATG-3′	95°C 10 sec, 95°C 5 sec, 60°C 20 sec×44 cycles
	anti-sense-Q	5′-TGGGTTGGGAGGTTGCTTC-3′	
hDJ-1	sense-Q	5′-GGTGCAGGCTTGTAAACATATAAC-3′	95°C 10 sec, 95°C 5 sec, 60°C 20 sec×44 cycles
	anti-sense-Q	5′-CTCTAAGTGATCGTCGCAGTTCGC-3′	
hACTB	sense-Q	5′-CCGACAGGATGCAGAAGCAG-3′	95°C 10 sec, 95°C 5 sec, 60°C 20 sec×44 cycles
	anti-sense-Q	5′-GTGGGGTGGCTTTTAGGATG-3′	

R: RT-PCR; Q: Real-time PCR.

### Luciferase Activity

pGL3-CCK-1615 was kindly provided by Dr. T Yoshikawa [27]. Nucleotide sequences of oligonucleotides used for PCR primers to construct deletion mutants of the promoter are as follows: CCK-Luc reverse: 5′-AGATCTGAGGACCAGCGGGC-3′, CCK-d1∶5′-GGTACCGAGTTGAGGTCCCAA-3′, CCK-d2∶5′-GGTACCCAGGAAAGGTGCGA-3′, CCK-d3∶5′-GGTACCATCACGCCTTGCTGA-3′, CCK-d4∶5′-GGTACCTCTTTTTCGCAACTGAAC-3′, CCK-d5∶5′-GGTACCGTTGCAGAAACGCAGC-3′, CCK-d6∶5′-GGTACCCCACCCTCACACTG-3′, CCK-d7∶5′-GGTACCGCAGCAACCGGAG-3′ and CCK-d8∶5′-GGTACCCGCCCCCCCAA-3′. PCR products were digested with KpnI and BglII and inserted into KpnI and BglII sites of pGL3-Basic (Promega, Madison, WI, USA). NIH3T3 and D2 cells in 24-well dishes were transfected with 0.5 µg of pGL3-CCK-1615 or its deletion reporter plasmids and various amounts (0–1.0 µg) of pEF-DJ-1-HA together with 0.25 µg of pCMV-β-gal by the calcium phosphate method. At 44 hrs after transfection, whole cell extract was prepared by addition of Triton X-100-containing solution from the Pica gene kit (Wako Pure Chemicals, Osaka, Japan) to the cells. About a one-fifth volume of the extract was used for the β-galactosidase assay to normalize the transfection efficiencies as described previously [6], and the luciferase activity due to the reporter plasmid was determined using a luminometer (Luminocounter Lumat LB 9507, EG & G Berthold, Bad Wildbad, Germany). Proteins in aliquots of the cell extract were analyzed by Western blotting with an anti-HA antibody (1∶2000, MBL, Nagoya Japan). Proteins on the membrane were reacted with an Alexa Fluor 680-conjugated secondary antibody (Molecular Probes, Eugene, OR, USA) and visualized by using an infrared imaging system (Odyssey, LI-COR, Lincoln, NE, USA).

### Chromatin Immunoprecipitation (ChIP) Assay

ChIP assays using cultured SH-SY5Y cells were performed according to the protocol of the ChIP Assay Kit (Millipore, Billerica, MA, USA). Briefly, after proteins had been cross-linked with DNA, cell pellets were resuspended in an SDS-lysis buffer and sonicated on ice using a sonicator (UR-20P, TOMY, Tokyo, Japan) 3 times for 20 sec each time. Genomic DNA was sheared to 300 to 1200 base pairs of length. Chromatin solution from 1×10^6^ cells/dish was preincubated with salmon sperm DNA and Protein A-agarose and incubated with species-matched IgG or with specific antibodies overnight at 4°C. DNA fragments immunoprecipitated were then used as templates for PCR with Ex taq (TaKaRa Bio, Kyoto, Japan) and reacted for 1 min at 94°C, 0.5 min at 90°C, 0.5 min at 56°C and 30 cycles of 30 sec at 72°C. Nucleotide sequences of oligonucleotides used for PCR primers were as follows: hCCK-1579/-1558∶5′-CTGGGGGTATTTCGATAACAAG-3′, hCCK-1291/-1306∶5′-TGTGGCAACGCCAGTGTG-3′, hCCK-302/-285∶5′-CACGCCTTGCTGATGCTC-3′ and hCCK-14/-32∶5′-AGGGGGAGGTGGTCTAGTG-3′. PCR products were separated on a 2% agarose gel and stained with ethidium bromide. Antibodies used were anti-RREB1 (1∶500, LifeSpan Biosciences, Seattle, WA, USA), anti-Sp1 (1∶500, Millipore) and anti-DJ-1 (1∶4000) antibodies. Rabbit anti-DJ-1 antibody was established by us as described previously [1] and affinity-purified using a DJ-1-coupled sepharose bead.

### Co-immunoprecipitation Assay

Proteins were extracted from cultured cells by the procedure described previously [6]. The proteins were immunoprecipitated with an anti-RREB1 antibody (1∶500), anti-DJ-1 antibody (1∶500) and anti-Sp1 antibody (1∶500) or normal IgG, and precipitates were analyzed by Western blotting with anti-RREB1 (1∶1000), anti-Sp1 (1∶1000) or mouse anti-DJ-1 antibody (1∶1000, 3E8, MBL). Proteins on membranes were visualized as described above.

### Electric Mobility Shift Assay (EMSA)

EMSA using IRDye800-conjugated probes was carried out as described previously [26]. For an experiment with specific antibodies, the nuclear extract was first incubated with the IRDye800-conjugated probe and then incubated with 2 µg of anti-DJ-1, anti-RREB1 and anti-Sp1antibodies or non-specific IgG for 30 min at 4°C. Nucleotide sequences of upper strand oligonucleotides for probes were as follows: hRRE: 5′- GCCGCCCCCCCAACCCCCCCACCCCGCCTC-3′ and hRREm: 5′- GCCGCCCCCCCGGCCCCCCCACCCCTCTTC-3′.

### Measurement of the CCK Level in DJ-1-knockout Mice

Wild-type and DJ-1-knockout mice were described previously [16]. DJ-1-knockout mice with C57BL/6 background and C57BL/6 mice that were used as control mice with *DJ-1* (+/+) were housed under the SPF condition, and all of the mice were basically fed with normal diet (D12337, Research Diets, Inc. New Brunswick, NJ). Serum was isolated from wild-type and DJ-1-knockout mice at 23 weeks of age. One hundred µl of serum was mixed with 100 µl of methanol/acetonitrile (1∶1 in volume) and centrifuged at 13,000 rpm at 4°C. The supernatant was then filtrated through a nitrocellulose membrane with 0.4 µm pore size and its amount of CCK was measured using an LC-MS (Xevo G2 QTof, Waters, Milford, MA, USA) and a software (TergetLynx™, Waters).

### Statistical Analyses

Data are expressed as means ± S.D. Statistical analyses were performed using analysis of variance (one-way ANOVA) followed by unpaired Student’s *t*-test or the Tukey-Kramer test.

### Ethics Statement

All animal experiments were carried out in accordance with the National Institutes of Health Guide for the Care and Use of Laboratory Animals, and the protocols were approved by the Committee for Animal Research at Hokkaido University (the permit number 08–0467).

## Results

### Reduced Expression of Cholecystokinin Gene in DJ-1-knockdown Cells

**Figure 1:**
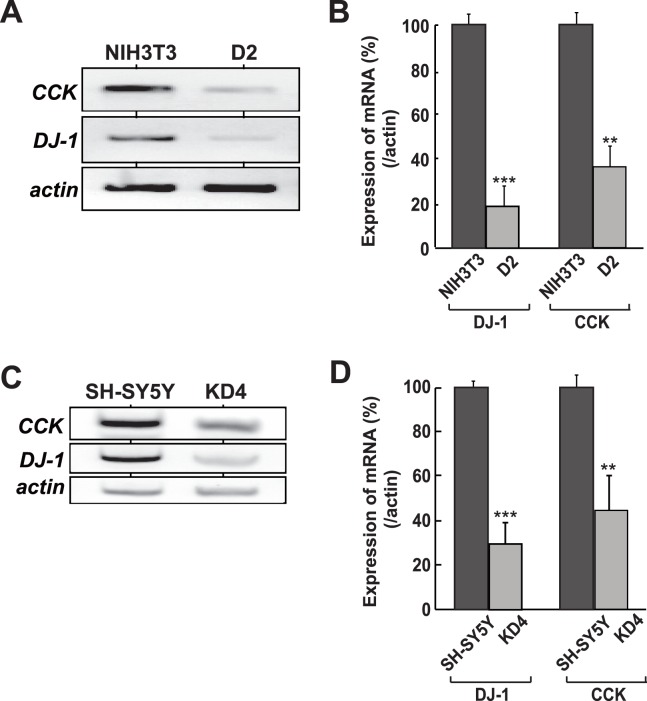
Reduction of *CCK* gene expression in DJ-1-knockdown cells. A and C. Relative mRNA levels of CCK and DJ-1 were examined by semi-quantitative RT-PCR in NIH3T3 and its DJ-1-knockdown D2 cells (A) and in SH-SY5Y and its DJ-1-knockdown KD4 cells (C). Actin mRNA was also amplified by PCR as loading controls. Reverse images of black and white staining are shown. B and D. Relative mRNA levels of CCK and DJ-1 were examined by quantitative RT-PCR (real-time PCR) in NIH3T3 and its DJ-1-knockdown D2 cells (B) and in SH-SY5Y and its DJ-1-knockdown KD4 cells (D). Actin mRNA was also amplified by real-time PCR as loading controls. All of the experiments were carried out 4 times (n = 4).

We have screened genes whose expression was reduced in D2 cells, which are DJ-1-knocked down mouse NIH3T3 cells, compared to that in parental NIH3T3 cells by using a DNA microarray, and the cholecystokinin (CCK) gene was found to be a candidate gene [17]. To confirm this, total RNA was extracted from D2 and NIH3T3 cells and the expression levels of CCK, DJ-1 and actin mRNA were examined by semi-quantitative RT-PCR. Actin mRNA was used as a loading control. As shown in Fig. 1A, the expression levels of CCK and DJ-1 mRNAs in D2 cells were reduced to about 20% of that in NIH3T3 cells. A reduced expression level of CCK mRNA was also observed in human SH-SY5Y and its knockdown KD4 cells, in which the expression level of CCK mRNA was reduced to 30% of that in parental SH-SY5Y cells (Fig. 1C). Reduced levels of CCK mRNA in DJ-1-knockdown cells were further confirmed by quantitative RT-PCR (real-time PCR) (Figs. 1B and 1D). These results indicate that reduced expression of DJ-1 rendered reduced expression of the *CCK* gene.

### Stimulation of CCK Promoter Activity by DJ-1

**Figure 2:**
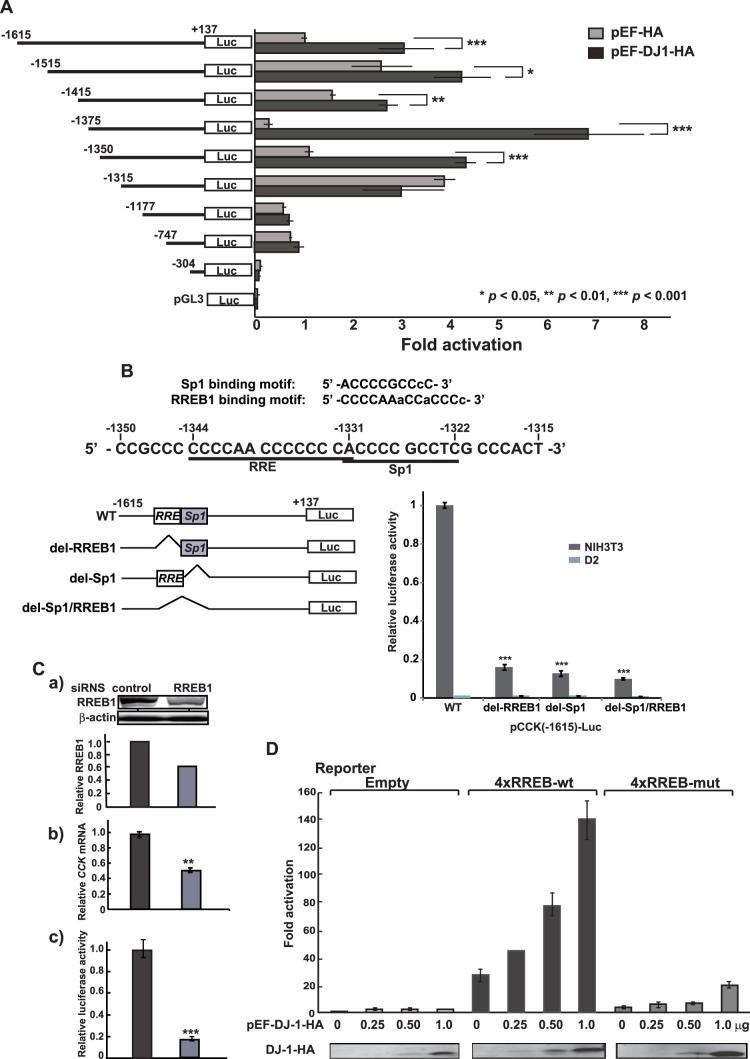
Stimulation of promoter activity of the *CCK* gene by DJ-1. A. Various deletion constructs of the CCK promoter linked to the *luciferase* gene were constructed and transfected into D2 cells together with pEF or pEF-DJ-1-HA. Forty-four hrs after transfection, cell extracts were prepared and their luciferase activity was measured as described in Materials and methods. n = 6. B. pGL3-CCK-1615 (WT) and reporter constructs of pGL3-CCK-1615 with deletion of recognition sites of either RREB1 (del- RREB1) or Sp1 (del-Sp1) and of both RREB1 and Sp1 (del-Sp1/RREB1) were transfected into NIH3T3 and D2 cells. Forty-four hrs after transfection, cell extracts were prepared and their luciferase activity was measured as described in Materials and methods. n = 4. C. NIH3T3 cells were transfected with RREB1 siRNA or with control siRNA. a) At 48 hrs after transfection, proteins were extracted from cells and analyzed by Western blotting with anti-RREB1 and anti-actin antibodies. b) At 48 hrs after transfection, total RNAs were extracted from cells, and the expression levels of *CCK* and *actin* mRNA were examined by real-time PCR, and relative expression of CCK versus actin is shown. c) At 24 hrs after siRNA transfection, pGL3-CCK-1615 (WT) was transfected into siRNA-transfected cells and their luciferase activities were measured at 48 hrs after transfection of pGL3-CCK-1615 (WT). n = 3. D. D2 cells in a 6-well dish were transfected with 0.75 µg of pSVP-Luc (Empty), pSVP-4xRREB1-wt (4xRREB1-wt) and pSVP-4xRREB1-mut (4xRREB1-mut) together with 0.25, 0.75 and 1.0 µg of pEF-DJ-1-HA. Forty-four hrs after transfection, cell extracts were prepared and their luciferase activity was measured. The expression level of DJ-1-HA in cell extracts was analyzed by Western blotting with an anti-HA antibody. n = 3.

To examine the effect of DJ-1 on CCK gene promoter activity, the upstream region of the *CCK* gene spanning −1615 to +137 linked to the *luciferase* gene [27], pGL3-CCK-1615, was transfected into D2 and NIH3T3 cells and its luciferase activity was measured. As shown in the first two lanes of Fig. 2B right panel, luciferase activity in D2 cells was reduced to 5% of that in NIH3T3 cells, suggesting that promoter activity of the *CCK* gene was attenuated in DJ-1-knockdown cells. To further assess the effect of DJ-1 on CCK promoter activity, D2 cells were transfected with pGL3-CCK-1615 together with expression vectors for DJ-1-HA and HA alone, and the luciferase activity was measured. The results showed that DJ-1-HA, but not HA, stimulated luciferase activity (Fig. 2A). To determine the region targeted by DJ-1, various deletion constructs of the CCK promoter linked to the *luciferase* gene were constructed and they were transfected into D2 cells with expression vectors for DJ-1-HA and HA alone. As shown in Fig. 2A, luciferase activities of various deletions up to −1350 from a transcriptional start site were similarly increased by DJ-1-HA but not by HA, and a deletion up to −1315 gave luciferase activities that were similarly reacted both with DJ-1-HA and HA, suggesting that the region −1350 to −1315 contains the DJ-1-responsive region. A computer search (TFSEARCH: (http://www.cbrc.jp/research/db/TFSEARCHJ.html) indicated that this region contains putative recognition sites for two transcription factors, Ras-responsive element-binding protein 1 (RREB1) and Sp1 (Fig. 2B). One RREB1-site (RRE) was found in this region. Since RREB1 plays a role in the Ras/Raf signaling cascade [28] and since *DJ-1* is an oncogene in cooperation with activated *ras* [1], the relationship between DJ-1 and RREB1 is interesting.

**Figure 3:**
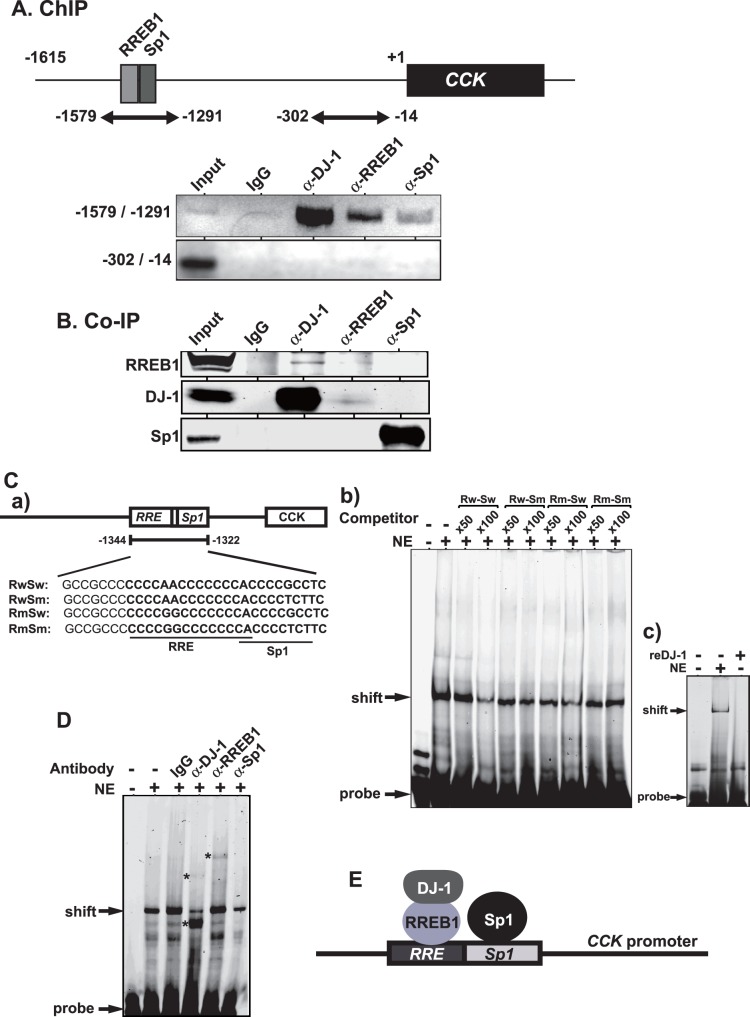
Association of DJ-1 with RREB1 on the LDLR promoter. A. Chromatin immunoprecipitation assays were carried out using chromatin prepared from SH-SY5Y cells. Chromatin was immunoprecipitated with anti-DJ-1, anti-RREB1 and anti-Sp1 antibodies or non-specific IgG. After extraction of DNA from precipitated chromatin, two regions spanning −1579 to −1291 and spanning −302 to −14 were amplified by PCR with specific primers and with amplified DNA and were separated on agarose gels as described in Materials and methods. n = 3. B. Proteins in SH-SY5Y nuclear extracts were immunoprecipitated with anti-RREB1, anti-DJ-1 and anti-Sp1 antibody or IgG. Immunoprecipitates were then analyzed by Western blotting with anti-RREB1, anti-DJ-1 and anti-Sp1 antibodies. n = 3. C. Nucleotide sequences used for labeled probe and competitors are shown (C-a). EMSA was carried out using SH-SY5Y cell nuclear extracts, IRDye800-labeled wild-type prove and non-labeled wild-type or mutated RRE competitors as described in Materials and methods (C-b). n = 3. MSA was carried out using SH-SY5Y cell nuclear extracts and 100 ng of recombinant DJ-1 on IRDye800-labeled wild-type prove (C-c). D. SH-SY5Y cell nuclear extract was first incubated with the IRDye800-conjugated probe and then incubated with 2 µg of anti-DJ-1, anti-RREB1 and anti-Sp1antibodies or non-specific IgG for 30 min at 4°C and subjected to EMSA. n = 3. E. Schematic model of DJ-1, RREB1 and Sp1 on the CCK promoter. DJ-1 complexed with RREB1 and Sp1 bind to the RRE and Sp1 site, respectively, to activate the CCK promoter.

To examine roles of RREB1 and Sp1 in activation of the CCK promoter, pGL3-CCK-1615 (wild-type) and reporter constructs of pGL3-CCK-1615 with deletion of recognition sites of either RREB1or Sp1 and of both RREB1 and Sp1 were transfected into NIH3T3 cells and their luciferase activities were measured. The results showed that luciferase activities of all of the deletion constructs were decreased to less than 20% of that of the wild-type construct in NIH3T3 cells (Fig. 2B), suggesting that both RREB1 and Sp1 are required for activation of CCK gene expression. Since DJ-1 was associated with RREB1 but not with Sp1 (Fig. 3B), we further examined the effect of RREB1 on CCK promoter activity and on CCK mRNA expression. NIH3T3 cells were transfected with siRNA targeting RREB1 (RREB1 siRNA) or with non-specific siRNA (control siRNA), and the expression level in RREB1 siRNA-transfected cells was found to be reduced to 60% of that in control siRNA-transfected cells by Western blotting (Fig. 2C-a). At 48 hrs after transfection, the CCK mRNA level was examined by real-time PCR and found to be reduced to 50% of that in control cells (Fig. 2C-b). Transfection of CCK promote-luciferase constructs into knockdown cells also showed reduced luciferase activity compared to that in control cells (Fig. 2C-c). These results indicate that RREB1 is a positive regulator for the CCK promoter. To further examine the effect of the RRE on CCK promoter activity, four oligonucleotides containing wild-type RRE and mutated RRE were ligated in tandem and then ligated with the SV40 promoter linked to the *luciferase* gene. These reporter constructs, p4xRREB-wt-Luc and p4xRREB-mut, and an SV40 promoter-luciferase construct (empty vector) were co-transfected with an expression vector for DJ-1-HA into D2 cells. As shown in Fig. 2D, luciferase activity of 4xRREB-wt, but not that of 4xRREB-mut or SV40 promoter, was increased by DJ-1-HA in a dose-dependent manner, indicating that RRE is a target for DJ-1.

### Association of DJ-1 with the Ras-responsive Element and RREB1

To examine the association of DJ-1 with the RRE, chromatin immunoprecipitation assays were carried out. Chromatin extracted from SH-SY5Y cells was reacted with non-specific IgG or anti-DJ-1, anti-RREB1 and anti-Sp1 antibodies, and two regions spanning −1579 to −1291 and spanning −302 to −14 were amplified by PCR with specific primers and with precipitated DNA as a template. As shown in Fig. 3A, anti-DJ-1, anti-RREB1 and anti-Sp1 antibodies, but not IgG, precipitated the region spanning −1579 to −1291, and no amplification in the region spanning −302 to −14 was observed, indicating that DJ-1, RREB1 and Sp1 bound to this region. Since it has been reported that DJ-1 does not directly bind to DNA [16] but since it has been reported that RREB1 and Sp1 directly bind to the RRE and Sp1 sites, respectively, it is possible that DJ-1 binds to the RRE or Sp1 site in association with RREB1 or Sp1. To examine this possibility, proteins in SH-SY5Y cell extracts were immunoprecipitated with anti-RREB1, anti-DJ-1 and anti-Sp1 antibodies or IgG, and precipitates were analyzed by Western blotting with anti-RREB1, anti-DJ-1 and anti-Sp1 antibodies. As shown in Fig. 3B, the anti-RREB1 antibody precipitated DJ-1 but not Sp1, the anti-DJ-1 antibody precipitated RREB1 but not Sp1 and the anti-Sp1 antibody precipitated neither DJ-1 nor RREB1, indicating association of DJ-1 with RREB1 and suggesting independent binding of DJ-1/RREB1 and Sp1 to RRE and Sp1 sites. To examine this possibility, gel-mobility shift assays were carried out using SH-SY5Y cell extracts and IRDye 800-labeled 23 bases of an oligonucleotide containing RRE and Sp1 sites (Fig. 3C-a). Competition experiments using 50 and 100-times molar ratio of non-labeled oligonucleotides with wild-type and mutated RRE or Sp1 sequences compared to that of labeled oligonucleotide showed that intensities of a shifted band containing proteins and DNA were reduced by wild-type RRE and Sp1but not by mutated RRE and Sp1 sequences (Fig. 3C-b). No direct binding activity of DJ-1 to the RRE was conformed using recombinant DJ-1 (Fig. 3C-c). Furthermore, SH-SY5Y cell extracts were first reacted with non-specific IgG or anti-DJ-1, anti-RREB1 and anti-Sp1 antibodies, and then gel-mobility-shift assays were carried out. As shown in Fig. 3D, shifted bands were further shifted slightly up and largely down after addition of an anti-DJ-1 antibody. A supershifted band was observed after addition of an anti-RREB1 antibody and the intensity of shifted band was reduced by an anti-Sp1 antibody. These results indicate that the DJ-1/RREB1 complex and Sp1 separately bind to RRE and Sp1 sites on the CCK promoter (Fig. 3E).

**Figure 4:**
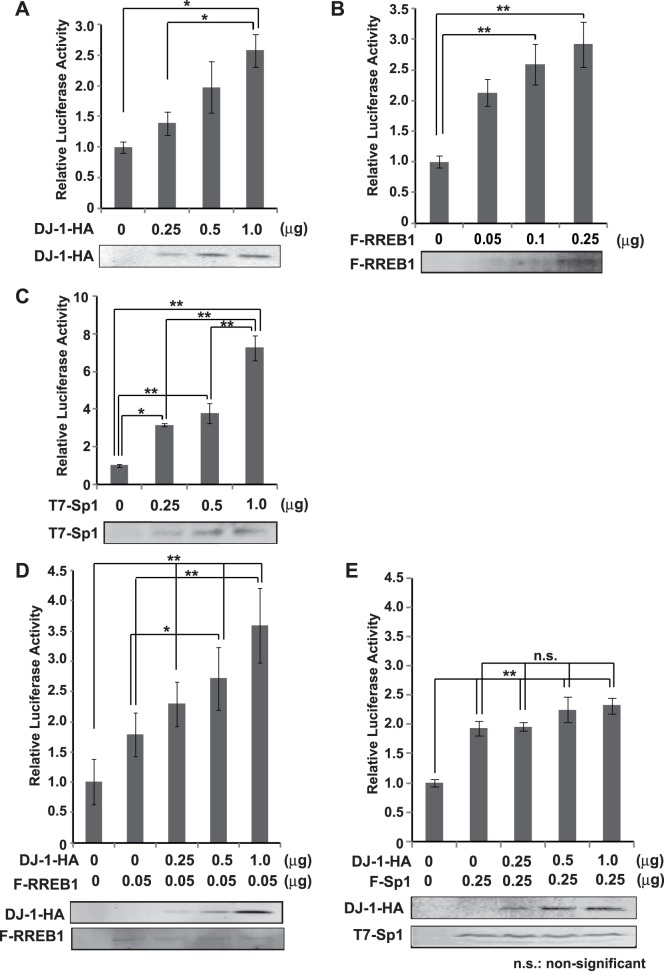
Stimulation of CCK promoter activity by DJ-1 along with RREB1. A–C. D2 cells were transfected with various amounts of expression vectors for DJ-1-HA (A), FLAG-RREB1 (B) and T7-Sp1 (C) along with pGL3-CCK-1615. Forty-eight hrs after transfection, cell extracts were prepared and their luciferase activity was measured as described in Materials and methods. n = 4. D and E. D2 cells were transfected with various amounts of expression vectors for DJ-1-HA along with fixed amount of FLAG-RREB1 (D) or T7-Sp1 (E) and pGL3-CCK-1615. Forty-eight hrs after transfection, cell extracts were prepared and their luciferase activity was measured as described in Materials and methods. n = 4. Statistical analyses were performed using the Tukey-Kramer test.

To examine a role of the DJ-1/RREB1 complex and Sp1 in CCK promoter activity, D2 cells were transfected with pGL3-CCK-1615 along with various amounts of an expression vector for DJ-1-HA, FLAG-RREB1 or T7-Sp1 and their luciferase activity was measured. The results showed that DJ-1-HA, FLAG-RREB1 and T7-Sp1 stimulated luciferase activity in a dose-dependent manner (Figs. 4A–4C). When various amounts of the expression vector for DJ-1-HA were transfected into D2 cells along with fixed amount of the expression vector for FLAG-RREB1 or T7-Sp1, DJ-1-HA stimulated luciferase activity in the dose-dependent manner only in the combination with FLAG-RREB1 but not with T7-Sp1 (Figs. 4D and 4E). These results support complex formation of DJ-1 with RREB1 but not with Sp1.

### The Reduced CCK Level in the Serum from DJ-1-knockout Mice

**Figure 5:**
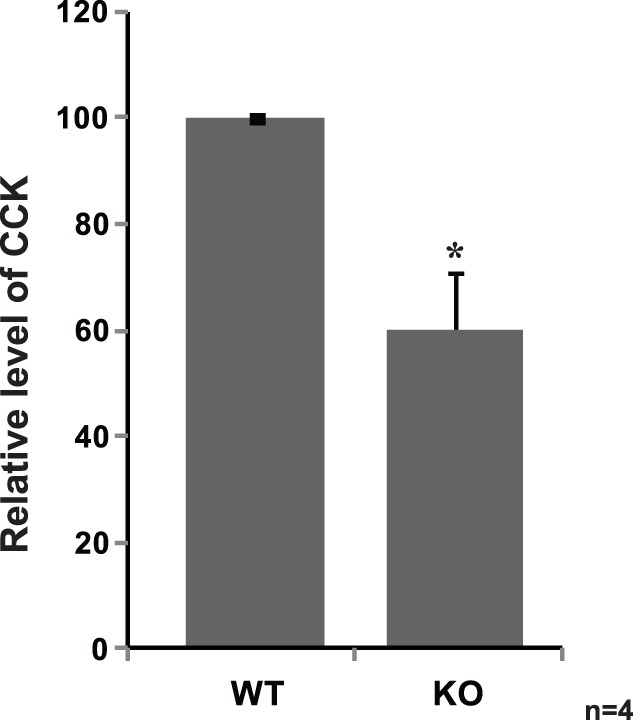
Reduced CCK level in the serum from DJ-1 knockout mice. Serum was isolated from wild-type and DJ-1-knockout mice at 23 weeks of age and the amount of CCK in serum was measured using an LC-MS as described in Materials and methods. Average amounts of CCK in wild-type and DJ-1-knockout mice are 21.9 and 14.6 µM, respectively. n = 4.

Since DJ-1 regulates the *CCK* expression as described above, the effect of DJ-1 on CCK levels was examined using DJ-1-knockout mice. As shown in Fig. 5, the total CCK amounts in serum from DJ-1-knockout mice at 23 weeks of age were significantly reduced to about 58% of those from wild-type mice at 23 weeks of age.

## Discussion

In this study, we found that DJ-1 positively regulates *CCK* gene expression at the transcriptional level through association of RREB1 on the RRE located in the CCK promoter. Reduced expression of the *CCK* gene was observed in two lines of DJ-1-knockdown cells (Fig. 1), and the reduced CCK level was found in the serum from DJ-1-knockout mice compared to that in wild-type mice (Fig. 5). This is the first finding of participation of DJ-1 in regulation of a peptide hormone gene. Deletion and point mutation analysis of the CCK promoter showed that two elements, RRE and Sp1 sites, were important for CCK promoter activity and that the RRE was a target for DJ-1 (Fig. 2). Although a chromatin immunoprecipitation (ChIP) assay using SH-SY5Y chromatin showed that both anti-RREB1 and anti-Sp1 antibodies in addition to an anti-DJ-1 antibody precipitated the regions containing RRE and Sp1 sites, co-immunoprecipitation assays showed that DJ-1 associated with RREB1 but not with Sp1 (Figs. 3A and 3B, respectively). These results suggest that the DJ-1/RREB1 complex and Sp1 independently recognize RRE and Sp1 sites, respectively, but that RREB1 and Sp1 mutually regulate expression of the *CCK* gene (Fig. 3E). Indeed, DJ-1 stimulated CCK promoter activity along with RREB1 but not with Sp1 (Fig. 4).

RREB1 is a ubiquitously expressed zinc finger protein [28] that represses several other promoters such as p16 [29], the prostate-specific antigen [30] and zeta-globin [31] through binding to the RREB1 sites in these promoters. RREB1 is a negative coregulator of the androgen receptor and its repressive activity toward AR-regulated genes is attenuated by activated Ras [30], [32]. DJ-1, on the other hand, binds to the androgen receptor (AR) complex, including PIASxα and DJBP, [6], [7], and positively regulates the expression of AR-regulated genes. DJ-1 is a ras-dependent oncogene product [1] and activates the ERK signaling pathway by an unknown mechanism [33]. RREB1 also plays a role in the Ras/Raf signaling pathway [28], [30], [32]. It is therefore thought that DJ-1 and RREB1 as a complex participate in positive or negative regulation of transcription factors related to Ras/Raf signaling.

CCK is co-localized with dopamine in the substantia nigra to regulate release of dopamine [20]–[23]. DJ-1 regulates expression of dopamine-synthesizing enzymes, tyrosine hydroxylase (TH) and L-DOPA decarboxylase (DDC) and of a dopamine- transporting enzyme into synaptic vesicles, vesicular monoamine transporter 2 (VMAT2) by activation of TH and VMAT2 genes and by interaction with TH, DDC and VMAT2 [9], [12], [34], [35]. These results and findings in this study suggest that DJ-1 directly or indirectly regulates synthesis and secretion of dopamine.
